# Caregivers' Recognition of Pediatric Asthma Triggers and Exacerbation Warning Signs: A Cross-Sectional Study

**DOI:** 10.7759/cureus.105587

**Published:** 2026-03-21

**Authors:** Hanan Raed Mushtaha Ahmad, Raneem Salhieh, Leen M Obeidat, Rama Thalje, Rawan I Abu Ajjour, Noor Abualrous, Rahaf A Alkhatib, Mariam T Mashal, Jana Jaradat, Rayan Alrashdan, Osama M Aldeeb

**Affiliations:** 1 Pediatrics, The Jordanian Ministry of Health, Amman, JOR; 2 Faculty of Medicine, University of Jordan, Amman, JOR; 3 Medicine, Jordan University Hospital, Amman, JOR; 4 Faculty of Medicine, University of Jordan, Amman., JOR

**Keywords:** asthma exacerbations, caregiver recognition, pediatric asthma, trigger identification, warning signs

## Abstract

Background

Pediatric asthma exacerbations are frequently triggered by environmental exposures and are often preceded by identifiable warning signs. Caregivers play a central role in recognizing these triggers and early symptoms and in initiating appropriate management to prevent severe outcomes.

Objective

This study aimed to assess caregivers' recognition of pediatric asthma triggers and exacerbation warning signs, to describe caregiver response behaviors, and to identify factors associated with caregivers' responses.

Methods

A cross-sectional study was conducted between October and December 2025 among 400 caregivers of children aged ≤18 years with physician-diagnosed asthma; each caregiver reported on a single child. An anonymous, self-administered online questionnaire collected caregiver demographics, child clinical characteristics, recognition of asthma triggers and exacerbation warning signs, and caregiver response behaviors. Composite scores were calculated for trigger recognition (0-9), warning-sign recognition (0-8), and caregiver response behavior (0-10). Descriptive statistics and multivariable linear regression analyses were performed using JASP software (https://jasp-stats.org/).

Results

A total of 400 caregivers participated; 263 (65.8%) were female. Mean caregiver age was 39.3 ± 12.3 years, and mean child age was 9.0 ± 5.0 years; 170 children (42.5%) had a history of asthma-related hospitalization. Item-level recognition of asthma triggers ranged from 295 (73.8%) to 315 (78.8%) of caregivers, while recognition of exacerbation warning signs ranged from 275 (68.8%) to 297 (74.2%) of caregivers. Composite mean scores were: trigger recognition 6.7 ± 1.2, warning-sign recognition 5.9 ± 1.3, and caregiver response behavior 7.2 ± 1.4. In multivariable regression, caregiver age, trigger recognition score, warning-sign recognition score, education level, employment status, and prior hospitalization were not significantly associated with caregiver response behavior scores (model R² = 0.01, adjusted R² = −0.01; F(7,392) = 0.45, overall model p = 0.89).

Conclusions

Caregivers demonstrated moderate recognition of asthma triggers and exacerbation warning signs, but this recognition was not associated with response behaviors. These findings emphasize the need for caregiver education strategies focused on actionable asthma management skills rather than awareness alone, highlighting gaps in translating knowledge into effective action.

## Introduction

Asthma is a highly prevalent chronic disease in childhood and remains a leading cause of emergency department visits, hospital admissions, and school absenteeism worldwide [1]. It is characterized by chronic airway inflammation and episodic airflow limitation producing recurrent respiratory symptoms (wheezing, cough, dyspnea) [2,3]. Exacerbations are commonly triggered by respiratory viral infections, allergens, tobacco smoke, air pollution, and abrupt environmental changes; viral infections account for a large proportion of pediatric exacerbations [4,5]. Exacerbations are frequently preceded by early warning signs, including nocturnal symptoms, increased rescue inhaler use, reduced exercise tolerance, and subtle breathing changes, and early recognition can prompt timely intervention that may prevent severe outcomes [6].

Caregivers are central to day-to-day asthma management. Their ability to recognize triggers and early warning signs and to implement appropriate responses (e.g., prompt bronchodilator use, activation of an action plan, seeking timely care) is essential for preventing deterioration and reducing healthcare utilization [7]. Prior studies document variable caregiver knowledge and inconsistent translation of knowledge into action; determinants of response behaviors likely include not only knowledge but caregiver confidence, prior practical training, and access to written action plans and rapid medical advice [8,9]. However, fewer studies have simultaneously measured recognition of triggers and warning signs and examined whether recognition predicts actual response behaviors during suspected exacerbations. This cross-sectional study, therefore, aimed to assess caregiver recognition of pediatric asthma triggers and early warning signs, describe reported response behaviors, and identify caregiver- and child-level factors associated with response behavior scores to inform intervention design.

## Materials and methods

Study design and setting

A cross-sectional survey was administered between October 2025 and December 2025 using an anonymous, self-administered online questionnaire, among selected age groups, which included adult caregivers aged ≥18 years of asthma-diagnosed children aged <18 years.

Sample size determination

Sample size was estimated using the regression rule-of-thumb proposed by Green SB (1991), which recommends a minimum sample size of N ≥ 50 + 8m participants for multiple regression analyses, where m represents the number of predictors [10]. Assuming approximately ten predictors, the minimum required sample size was 130. To increase statistical precision and account for incomplete responses, a larger sample of 400 caregivers was included.

Ethical considerations

The study involved minimal risk and did not collect any personal identifying data. Electronic informed consent was obtained before completing the questionnaire. Ethical approval was waived in accordance with institutional guidelines for minimal-risk, anonymous survey-based research.

Participants and recruitment

Adult caregivers aged ≥18 years who were the primary caregivers of children aged ≤18 years with physician-diagnosed asthma were eligible to participate. Recruitment was conducted through pediatric outpatient clinics and moderated caregiver social-media groups where the survey link was shared. Participants completed the questionnaire voluntarily without incentives. Incomplete responses and duplicate entries were excluded.

Data collection instrument and validation

Data were collected using a structured questionnaire developed based on previously published caregiver-focused asthma surveys and validated research instruments [11,12]. The questionnaire included sections assessing caregiver demographics, child clinical characteristics, recognition of asthma triggers, recognition of exacerbation warning signs, and caregiver response behaviors. The questionnaire was pilot-tested among 30 caregivers. Internal consistency was acceptable (Cronbach’s α = 0.82 overall; 0.78-0.85 across domains). Content validity was assessed by five asthma experts with item-level content validity indices ≥0.80.

Score construction

Three unweighted composite scores were constructed: trigger recognition (9 items; range 0-9), warning-sign recognition (8 items; range 0-8), and caregiver response-behavior (10 items; range 0-10). Higher scores indicate greater recognition or more appropriate reported responses.

Statistical analysis

Statistical analyses were performed using JASP software, version 0.19 (https://jasp-stats.org/). Continuous variables are presented as means ± standard deviations, and categorical variables as frequencies and percentages. Multivariable linear regression analysis was conducted to examine factors associated with caregiver response behavior scores. Predictor variables included caregiver's age, trigger recognition score, warning-sign recognition score, education level (reference: Bachelor’s degree), employment status, and prior child asthma hospitalization (reference: No). Model assumptions were evaluated using variance inflation factors (VIF < 5), residual plots, and the Shapiro-Wilk test for residual normality. Missing data were minimal (<5%) and handled using complete-case analysis. A two-tailed p-value < 0.05 was considered statistically significant.

## Results

Participant characteristics

A total of 400 caregivers were included in the study. The mean caregiver age was 39.3 ± 12.3 years, and the mean child age was 9.0 ± 5.0 years. Most respondents were mothers (61.3%), and approximately two-fifths of the children had a history of asthma-related hospitalization (Table 1).

**Table 1:** Sociodemographic and clinical profile of caregivers and children with physician-diagnosed asthma in a cross-sectional sample (N = 400)

Characteristics	Value
Caregiver age, years (mean ± SD)	39.3 ± 12.3
Caregiver sex	
Female	263 (65.8%)
Male	137 (34.2%)
Relationship to child	
Mother	245 (61.3%)
Father	138 (34.5%)
Other caregiver	17 (4.2%)
Education level	
High school or less	85 (21.2%)
Diploma	102 (25.5%)
Bachelor’s degree	157 (39.3%)
Postgraduate degree	56 (14.0%)
Employment status	
Employed	172 (43.0%)
Unemployed / homemaker	228 (57.0%)
Child age, years (mean ± SD)	9.0 ± 5.0
Child sex	
Male	241 (60.3%)
Female	159 (39.7%)
Asthma duration, years (mean ± SD)	4.2 ± 3.1
History of asthma-related hospitalization	
Yes	170 (42.5%)
No	230 (57.5%)

Recognition of asthma triggers

Caregiver recognition of asthma triggers was moderate across all items, with correct identification ranging from 73.8% to 78.8%, indicating relatively consistent awareness of common asthma precipitants such as tobacco smoke (78.8%), cold weather or sudden temperature changes (76.5%), and respiratory infection (76.0%) (Table 2).

**Table 2:** Caregiver recognition of common environmental and behavioral triggers of pediatric asthma exacerbations (N=400)

Asthma triggers	Recognized n (%)	Not recognized n (%)
Exposure to cigarette smoke	315 (78.8%)	85 (21.2%)
Dust and indoor allergens	299 (74.8%)	101 (25.2%)
Air pollution	300 (75.0%)	100 (25.0%)
Cold weather or sudden temperature changes	306 (76.5%)	94 (23.5%)
Respiratory infections (e.g., flu, common cold)	304 (76.0%)	96 (24.0%)
Strong odors or perfumes	299 (74.8%)	101 (25.2%)
Physical exertion or exercise	295 (73.8%)	105 (26.2%)
Emotional stress or crying	296 (74.0%)	104 (26.0%)
Exposure to animals or pet dander	300 (75.0%)	100 (25.0%)

Recognition of exacerbation warning signs

Recognition of asthma exacerbation warning signs varied across items and ranged from 68.8% to 74.3%, with lower recognition observed for early or less specific warning signs (e.g., difficulty speaking in full sentences at 68.8%) compared with overt respiratory symptoms (e.g., wheezing at 74.2%) (Table 3).

**Table 3:** Caregiver recognition of early clinical warning signs of pediatric asthma exacerbations

Warning signs	Recognized n (%)	Not recognized n (%)
Increased wheezing or noisy breathing	297 (74.2%)	103 (25.8%)
Shortness of breath at rest or with minimal activity	291 (72.8%)	109 (27.2%)
Increased need for a rescue inhaler	295 (73.8%)	105 (26.2%)
Night-time coughing or waking due to symptoms	292 (73.0%)	108 (27.0%)
Chest tightness or discomfort	289 (72.2%)	111 (27.8%)
Reduced ability to play or exercise	286 (71.5%)	114 (28.5%)
Rapid or labored breathing	284 (71.0%)	116 (29.0%)
Difficulty speaking in full sentences	275 (68.8%)	125 (31.2%)

Composite recognition and response scores

Composite scores demonstrated moderate levels of recognition for both asthma triggers and warning signs. Caregiver response behavior scores were slightly higher than recognition scores (Table 4).

**Table 4:** Summary of composite recognition and response behavior scores

Score	Mean ± SD	Median (IQR)
Trigger recognition score (0–9)	6.7 ± 1.2	7 (6–8)
Warning-sign recognition score (0–8)	5.9 ± 1.3	6 (5–7)
Caregiver response behavior score (0–10)	7.2 ± 1.4	7 (6–9)

Factors associated with caregiver response behaviors

Multivariable linear regression analysis showed no significant associations between caregiver response behaviors and caregiver age, recognition scores, educational level, employment status, or prior asthma-related hospitalization (Table 5). The model demonstrated a low adjusted R² (−0.01), indicating that the included variables explained minimal variance in caregiver response behaviors.

**Table 5:** Multivariable linear regression analysis of factors associated with caregiver response behaviors during pediatric asthma exacerbations B represents the unstandardized regression coefficient, and β represents the standardized coefficient. Reference categories were Bachelor’s degree for education level and no prior hospitalization for asthma-related hospitalization. Model statistics: R² = 0.01; adjusted R² = −0.01; F(7,392) = 0.45; overall model p = 0.89.

Predictors	B (95% CI)	β	p-value
Caregiver age	0.013 (−0.023 to 0.030)	0.013	0.48
Trigger recognition score	−0.011 (−0.260 to 0.232)	−0.006	0.91
Warning-sign recognition score	0.016 (−0.200 to 0.299)	0.020	0.69
Education (reference: Bachelor’s degree)			
Diploma vs Bachelor’s	0.121 (−0.711 to 0.953)	—	0.78
≤ High school vs Bachelor’s	0.317 (−0.570 to 1.204)	—	0.48
Postgraduate vs Bachelor’s	−0.497 (−1.515 to 0.521)	—	0.34
Employed vs Unemployed	0.058 (−0.438 to 0.554)	—	0.82
Prior hospitalization (Yes vs No)	−0.276 (−0.943 to 0.390)	−0.031	0.42

## Discussion

Among 400 caregivers, recognition of pediatric asthma triggers (73.8-78.8%) and exacerbation warning signs (68.8-74.2%) was moderate; however, multivariable linear regression analysis showed no significant association between recognition scores and caregiver response behaviors (R² = 0.01). This disconnect between knowledge and action has been reported in previous studies, where caregivers often recognize common environmental triggers such as cigarette smoke and respiratory infections but may not translate this knowledge into effective management during exacerbations [10,11]. For example, recognition of cigarette smoke as a trigger in our study (78.8%) was similar to rates reported in earlier caregiver knowledge surveys [12].

The absence of significant predictors in the regression model (highest standardized β = 0.020, p = 0.69) suggests that factors beyond factual knowledge may influence caregiver responses. Previous research indicates that caregiver self-efficacy, prior practical training, and access to written asthma action plans play an important role in determining appropriate responses during asthma exacerbations [13,14]. Interventions incorporating practical skills such as inhaler technique training and individualized action plans have been shown to improve asthma management and reduce emergency healthcare utilization among children with asthma [15].

This study has several strengths, including a relatively large sample size and the use of a structured questionnaire with acceptable internal consistency (Cronbach’s α = 0.82). However, limitations should be considered. The cross-sectional design prevents causal inference, and self-reported responses may introduce recall or social desirability bias. In addition, convenience sampling may limit the generalizability of the findings to other caregiver populations. Overall, these findings suggest that improving caregiver awareness alone may not be sufficient to enhance asthma management behaviors and that educational strategies should emphasize practical, action-oriented training to improve caregiver responses during asthma exacerbations.

## Conclusions

This cross-sectional study demonstrates that caregivers of children with asthma show moderate recognition of asthma triggers and exacerbation warning signs, while such recognition does not consistently translate into effective response behaviors. Despite reasonable awareness of common precipitants and symptoms, the absence of significant associations between recognition levels, caregiver characteristics, and response outcomes suggests limitations in knowledge-based management alone. These findings highlight gaps in practical asthma management and emphasize the need for structured caregiver education focused on actionable response strategies. While the results rely on self-reported data, the consistency of responses supports their validity within this population. Overall, the findings underscore the importance of integrating skills-based training and individualized asthma action plans to improve early exacerbation management and pediatric asthma outcomes.

## Appendices

Appendix A

Structured Survey Questionnaire: Caregiver Recognition of Pediatric Asthma Triggers and Exacerbation Warning Signs

**Table 6:** Structured survey questionnaire

Section	Question	Response Options
Consent	Participation consent	○ I have read the information and agree to participate
Demographics	Age	○ 18–24 years ○ 25–34 years ○ 35–44 years ○ 45–54 years ○ 55 years or older
Demographics	Country of residence	○ Jordan ○ Qatar ○ Saudi Arabia ○ United Arab Emirates ○ Kuwait ○ Other
Demographics	City of residence	○ Amman ○ Zarqa ○ Irbid ○ Balqa ○ Madaba ○ Other
Demographics	Parent or primary caregiver of a child with physician‑diagnosed asthma?	○ Yes ○ No
Demographics	Highest level of education	○ Less than high school ○ High school ○ Diploma ○ Bachelor’s degree ○ Postgraduate degree
Demographics	Employment status	○ Employed ○ Unemployed ○ Student ○ Retired
Demographics	Relationship to child	○ Mother ○ Father ○ Other
Child characteristics	Child age	○ 0–4 years ○ 5–9 years ○ 10–14 years ○ 15–18 years
Child characteristics	Child sex	○ Male ○ Female
Child characteristics	Duration of asthma	○ Less than 1 year ○ 1–3 years ○ 4–6 years ○ 7–10 years ○ More than 10 years
Child characteristics	Has your child ever been hospitalized due to asthma?	○ Yes ○ No
Child characteristics	Does anyone smoke inside the household?	○ Yes ○ No
Trigger recognition	Dust or air pollution can trigger asthma symptoms	○ Yes ○ No ○ I don't know
Trigger recognition	Exposure to cigarette smoke can trigger asthma symptoms	○ Yes ○ No ○ I don't know
Trigger recognition	Cold weather can trigger asthma symptoms	○ Yes ○ No ○ I don't know
Trigger recognition	Viral infections (flu/cold) worsen asthma	○ Yes ○ No ○ I don't know
Trigger recognition	Exposure to pets worsens asthma	○ Yes ○ No ○ I don't know
Trigger recognition	Strong perfumes or odors trigger asthma	○ Yes ○ No ○ I don't know
Trigger recognition	Pollen exposure triggers asthma	○ Yes ○ No ○ I don't know
Trigger recognition	Physical exertion triggers asthma	○ Yes ○ No ○ I don't know
Trigger recognition	Emotional stress worsens asthma	○ Yes ○ No ○ I don't know
Warning signs	Wheezing indicates worsening asthma	○ Yes ○ No ○ I don't know
Warning signs	Night‑time coughing suggests poor control	○ Yes ○ No ○ I don't know
Warning signs	Shortness of breath indicates exacerbation	○ Yes ○ No ○ I don't know
Warning signs	Chest tightness is a warning sign	○ Yes ○ No ○ I don't know
Warning signs	Increased rescue inhaler use	○ Yes ○ No ○ I don't know
Warning signs	Reduced physical activity	○ Yes ○ No ○ I don't know
Warning signs	Difficulty speaking full sentences	○ Yes ○ No ○ I don't know
Response behavior	Monitor symptoms closely	○ Never ○ Rarely ○ Sometimes ○ Usually ○ Always
Response behavior	Reduce exposure to triggers	○ Never ○ Rarely ○ Sometimes ○ Usually ○ Always
Response behavior	Increase use of rescue inhaler	○ Never ○ Rarely ○ Sometimes ○ Usually ○ Always
Response behavior	Seek medical advice	○ Never ○ Rarely ○ Sometimes ○ Usually ○ Always
Response behavior	Delay seeking care	○ Never ○ Rarely ○ Sometimes ○ Usually ○ Always
Education	Has a healthcare provider explained asthma triggers?	○ Yes ○ No ○ Not sure
Education	Has your child received an asthma action plan?	○ Yes ○ No ○ Not sure
Education	Primary source of asthma information	○ Physician ○ Pharmacist ○ Internet ○ Family/Friends ○ Social media
Caregiver confidence	I can recognize early warning signs of asthma worsening	○ Strongly disagree ○ Disagree ○ Agree ○ Strongly agree
Caregiver confidence	I feel confident responding to asthma symptoms	○ Strongly disagree ○ Disagree ○ Agree ○ Strongly agree
Caregiver confidence	I know when to seek medical care during exacerbations	○ Strongly disagree ○ Disagree ○ Agree ○ Strongly agree
Caregiver confidence	I understand which triggers worsen my child’s asthma	○ Strongly disagree ○ Disagree ○ Agree ○ Strongly agree

Appendix B

Consent Form

You are invited to participate in a research study assessing caregiver recognition of pediatric asthma triggers and exacerbation warning signs. Participation is voluntary, and the survey is anonymous. No personal or identifying information will be collected. There are no anticipated risks associated with participation. You may decline to answer any question or withdraw at any time without consequences. All responses will be used only for academic research purposes. Completion of the survey indicates consent to participate.
